# Buffalo Lithia Springs of Virginia

**Published:** 1890-06

**Authors:** Hunter McGuire

**Affiliations:** Richmond, Va.; Late Professor of Surgery, Medical College of Virginia


### BUFFALO LITHIA SPRINGS OF VIRGINIA.

USE OF THIS WATER AFTER LAPAROTOMY AND SURGICAL OPERATIONS GENERALLY.

HUNTER McGUIRE, M. D., LL. D.,
Late Professor of Surgery, Medical College of Virginia.

From the Virginia Medical Monthly for April, 1890.

RICHMOND, VA., March 22, 1890.

DEAR DR. EDWARDS—I have just received your letter of this date. I use Buffalo Lithia Water very freely in my Hospital. After every case of laparotomy, I give this water for its diuretic properties, and because the stomach bears it so well—often retaining it when everything else is rejected. Indeed, I use it freely after nearly all my surgical operations. It is especially valuable in supra-pubic cystotomy. Many years' experience in its use only confirms the good opinion I have so often expressed in regard to it. Yours, very truly,

HUNTER McGUIRE.

